# New Insights into the Consequences of Post-Windthrow Salvage Logging Revealed by Functional Structure of Saproxylic Beetles Assemblages

**DOI:** 10.1371/journal.pone.0101757

**Published:** 2014-07-22

**Authors:** Simon Thorn, Claus Bässler, Thomas Gottschalk, Torsten Hothorn, Heinz Bussler, Kenneth Raffa, Jörg Müller

**Affiliations:** 1 Sachgebiet Forschung und Dokumentation, Nationalparkverwaltung Bayerischer Wald, Grafenau, Germany; 2 Hochschule für Forstwirtschaft Rottenburg, Rottenburg am Neckar, Germany; 3 Abteilung Biostatistik, Universität Zürich, Zürich, Switzerland; 4 Bavarian State Institute for Forestry, Freising, Germany; 5 Department of Entomology, University of Wisconsin-Madison, Madison, United States of America; 6 Chair for Terrestrial Ecology, Department of Ecology and Ecosystem Management, Technische Universität München, Freising, Germany; Swiss Federal Institute for Forest, Switzerland

## Abstract

Windstorms, bark beetle outbreaks and fires are important natural disturbances in coniferous forests worldwide. Wind-thrown trees promote biodiversity and restoration within production forests, but also cause large economic losses due to bark beetle infestation and accelerated fungal decomposition. Such damaged trees are often removed by salvage logging, which leads to decreased biodiversity and thus increasingly evokes discussions between economists and ecologists about appropriate strategies. To reveal the reasons behind species loss after salvage logging, we used a functional approach based on four habitat-related ecological traits and focused on saproxylic beetles. We predicted that salvage logging would decrease functional diversity (measured as effect sizes of mean pairwise distances using null models) as well as mean values of beetle body size, wood diameter niche and canopy cover niche, but would increase decay stage niche. As expected, salvage logging caused a decrease in species richness, but led to an increase in functional diversity by altering the species composition from habitat-filtered assemblages toward random assemblages. Even though salvage logging removes tree trunks, the most negative effects were found for small and heliophilous species and for species specialized on wood of small diameter. Our results suggested that salvage logging disrupts the natural assembly process on windthrown trees and that negative ecological impacts are caused more by microclimate alteration of the dead-wood objects than by loss of resource amount. These insights underline the power of functional approaches to detect ecosystem responses to anthropogenic disturbance and form a basis for management decisions in conservation. To mitigate negative effects on saproxylic beetle diversity after windthrows, we recommend preserving single windthrown trees or at least their tops with exposed branches during salvage logging. Such an extension of the green-tree retention approach to windthrown trees will preserve natural succession and associated communities of disturbed spruce forests.

## Introduction

Forest ecosystems worldwide are periodically affected by natural disturbances, such as wind storms, fires, avalanches and insects [1], [2]. Since the 1990s, disturbance events in forests of the northern hemisphere have increased, particularly in mature conifer stands, owing to both an increase in growing stock and global climate change [3]–[5]. After such disturbances, forest managers try to limit the economic loss by focusing on saving downed wood from fungal infestation and avoiding an increase of pest species populations [6], [7]. Even if such salvage logging is broadly publicly accepted [8], both ecologists and conservationists are increasingly aware that natural disturbances conserve biodiversity in forests moulded by anthropogenic impacts [9], [10]. In addition, whether salvage logging should be conducted and how it would be best conducted, particularly in coniferous forests, is highly controversial [11]–[13].

Between 1950 and 2000 in Europe, wind storms annually damaged an estimated average of 18.7 million m^3^ of wood [3]. Such events often are followed by outbreaks of the European spruce bark beetle *Ips typographus* (Linnaeus, 1758), which damages an additional 2.9 million m^3^ wood annually [3]. In contrast to our knowledge of the high value of windthrows for biodiversity [14], [15], our knowledge about why particular species are affected or not by salvage logging is limited; most recent studies have focused on post-fire salvage logging [16]–[19] or economic consequences and bark beetles [20], [21]. The relatively few studies on post-windthrow salvage logging focus mainly on the decrease in species numbers [14], [22], [23].

Recent studies using, for example, guild-specific analysis of bird assemblages demonstrate that species richness poorly reflects the effect of human intervention [24]. A quantification of species loss is not sufficient to guide conservation efforts and resource management directly [25], [26]. Therefore, functional approaches have become increasingly important throughout broad areas of ecological research [26]–[28]. It has recently been proposed that the species position in a functional space can be used as a tool to reveal advanced warnings for changes in disturbed ecosystems [29].

Saproxylic beetles are highly diverse, play important roles in the decomposition of wood [30] and are sensitive to forest management, and are thereby an ideal model group to study the impact of salvage logging on biodiversity [31], [32]. Here we studied the impact of salvage logging and focused on recently published ecological traits of saproxylic beetles [33]: mean body size, diameter and decay stage of wood in which larvae develop and canopy cover of forests in which the species is known to occur.

The body size of saproxylic beetles is positively correlated to the diameter of the substrate used for larval development [34]. Assemblage values should therefore decrease with the removal of tree sections of large diameter [35], [36]. Similarly, a shift in assemblages toward species with a preference for wood of smaller diameters might follow the removal of major tree trunks. Since the cutting of uprooted trees in salvage-logging operations removes the trunk and the remaining branches rapidly decompose to advanced decomposition stages [37], we therefore expect that colonizers of earlier successional stages of wood decomposition also decline [35]. Finally, the removal of dead wood might decrease shady conditions provided by the cross-laminated arrangement of trees after windthrows, thereby promoting heliophilous species [38].

We calculated effect sizes of saproxylic beetle functional diversity, and mean assemblage values and diversity values of each single trait to test the predictions that salvage logging results in 1) a lower overall functional diversity, 2) lower mean body size and body size diversity, 3) a minor occurrence of species preferring dead wood of large diameter, 4) a decrease in species preferring early successional stages of decomposition and 5) an increase in species preferring open canopies.

## Methods

### Study area

The study was carried out in the high montane spruce forest in the Bavarian Forest National Park in south-eastern Germany. Forest stands in this area, at an elevation above 1,100 m, are naturally dominated by Norway spruce (*Picea abies*). Annual precipitation ranges from 1,300 to 1,800 mm, and annual mean air temperature ranges from 3.0 to 4.0°C [39].

**Figure 1:**
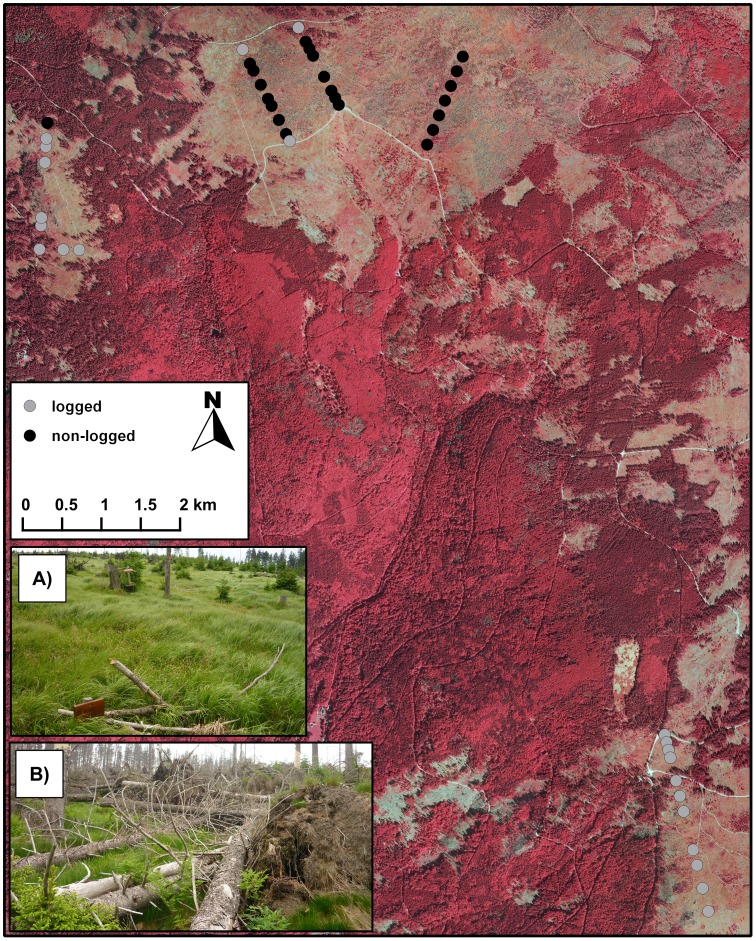
Locations of flight-interception traps within our study area in salvage-logged (A) and non-salvage-logged windthrows, sampled from 2008 to 2011 (B).

On January 16, 2007, an area of approximately 1,000 ha of spruce forests was felled to various extents by the windstorm Kyrill, ranging from single trees to stands covering several hundreds of hectares. From a total affected amount of wood of about 160,000 m^3^, 50,000 m^3^ are concentrated on four larger windthrow areas (∼170 ha). These centres were partially excluded from the overall salvage-logging operation. Such operations basically remove the main trunk to preserve it from fungal and pest species infestation. The branches are cut off the trunk and remain on the ground surface, which is covered by a grass layer of *Calamagrostis villosa* (32±11 cm height on logged plots and 32±9 cm height on non-logged plots, measured by relevés [39]). Standardized measurements of dead-wood objects per plot revealed that on salvaged-logged plots, 90% (n = 29) of dead-wood objects had direct contact with the ground surface, in contrast to only 10% (n = 35) on non-logged plots. Wind-felled trees on non-logged plots remained mostly alive after the storm in spring 2007 until they were colonized by *Ips typographus* in 2008. In contrast, salvage logging typically kill trees immediately. Salvage logging in the major windthrow areas removed about 255 m^3^/ha and was completed in autumn 2007 (Fig. 1).

### Beetle sampling

To reflect the emerging beetle fauna of surrounding dead wood, we used flight-interception traps [40]. Traps were established throughout windthrow centres and surrounding salvage-logged areas: 22 in logged areas and 22 in non-logged areas in spring 2008. Traps in logged areas were surrounded by at least a 50 m radius of completely salvage-logged windthrows (all trees removed); traps in non-logged areas were surrounded by at least a 50 m radius of completely non-logged windthrow (all trees of the previous stand were wind felled). Each trap consisted of a crossed pair of transparent plastic shields (40×60 cm) and contained a 3.0% copper-vitriol solution to preserve trapped specimens [41]. The shortest distance between two traps was 50 m, and the largest distance between traps was 6,500 m. Sampling was conducted during the entire growing season between May after the snow melted until September over four consecutive years until 2011. Traps were emptied monthly. All sampled beetles were identified to the species level, but only saproxylic beetles were considered in the analysis [42], [43].

### Trait characterization

We used four ecological traits that enabled us to link species habitat selection [33] directly to forest management: mean body size, diameter of wood in which the larvae of the species was recorded, decay stage of the wood, and canopy cover of forests in which the larvae of the species is known to occur. The single classes of niche positions were classified as follows: *wood diameter class*: 1, <15 cm; 2, 15–35 cm; 3, 35–70 cm; and 4, >70 cm; *wood decay stage*: 0, alive; 1, freshly dead (up to two years); 2, initiated decomposition with loose bark and tough sapwood; 3, advanced decomposition with soft sapwood and partly tough hardwood; and 4, extremely decomposed and mouldered; *canopy cover*: 1, open; 2, semi-open; and 3, closed (for mean niche positions of species, see Table S1).

### Data analysis

Prior to the main analysis, trapped specimens were grouped according to the trap level in each year, and subsequent analyses were conducted at the trap per year level. We calculated the mean trait value of the assemblage for each of the four single traits as an arithmetic mean, weighted by the number of trapped individuals (e.g. log-transformed number of individuals) per species. The results of abundance-weighted data, abundance-weighted data based on the log-transformed number of individuals, and presence/absence data were similar; therefore, we present only abundance-weighted results, which do not overestimate singletons and represents the majority of trapped beetles [40]. To test the impact of salvage logging on saproxylic beetles, we selected these mean assemblage trait values of every single trait and their related trait diversity as target variable and year, and we selected logged/non-logged as predictor variables.

A challenge in functional diversity measures is to compare dispersion of functional traits in the functional space independent of species numbers [29]. Hence, we calculated a distance matrix based on the pairwise Euclidean distance between functional traits of all possible species pairs within an assemblage per plot for each single trait [44]. Similarly, we used the function *dist* to calculate an overall distance matrix based on Euclidean distance between among four traits. We compared the observed value of mean niche position for each trait in each assemblage to an artificial assemblage with equal number of randomly selected species from the regional species pool (all species recorded on plots pooled). To create these random assemblages, we used null models with tip shuffling and abundance weighting in 999 randomizations using the function *ses.mpd* (abundance.weighted  = TRUE) in the add-on package *picante* of R version 2.15 [45], [46]. The effect size, which is provided by the null model, indicates the dispersion of a specific trait and compares it to the trait dispersion in an artificial assemblage. Values >0 indicate over-dispersion of a trait. In turn, values <0 indicate clustering and a trait dispersion in observed assemblages smaller than expected from a random assemblage. In general, over-dispersion is a result of competition or facilitation, and clustering is a result of environmental filtering [47].

The natural occurrence of windthrow patches necessitates that logged and non-logged plots are near each other in the same forest site (Fig. 1). This might reduce the independence of observations. Furthermore, we cannot treat the measurement on each plot as independent as the measurements were conducted in consecutive years. To address these issues, we analysed the data using a linear mixed random effects model with sampling plot and coordinates of sampling plot as random factors. The plot coordinates enable us to account for possible spatial autocorrelation within the arrangement of our plots, as a second-order trend surface (for the R code, see Text S1; for the method, see [48]).

To illustrate the impact of salvage logging on the primary target species, we modelled the mean number of *I. typographus* individuals per trap by using an observation-specific random factor to control the mixed model with Poisson distribution for potential over-dispersion [49]. To consider the resulting problem of testing hypothesis families, we applied a multiple post-hoc comparison with adjusted p-values using the function *glht* in the add-on package *multcomp* and constructed a matrix of coefficients [50]. This enabled us to compare the impact of salvage logging within each year and to compare changes within consecutive years on logged or non-logged plots.

## Results

### Effect of pest control

In total, 33,796 specimens belonging to 179 species of 37 saproxylic beetles families were sampled. Of these, 29 (89 individuals) species were found exclusively on non-logged plots, and 34 species (85 individuals) were found only on salvage-logged plots. Forty-two species (19 on non-logged plots) were caught as singletons. The most abundant species in our data on non-logged plots were *Ips typographus* (7,785 individuals), *Pityogenes chalcographus* (6,697 individuals) and *Xyloterus lineatus* (1,579 individuals), all of subfamily *Scolytinae* (Curculionidae). The most frequent species on logged plots were *P. chalcographus* (3,147 individuals), *Hylastes cunicularius* (1,101 individuals) and *I. typographus* (796 individuals).

**Figure 2:**
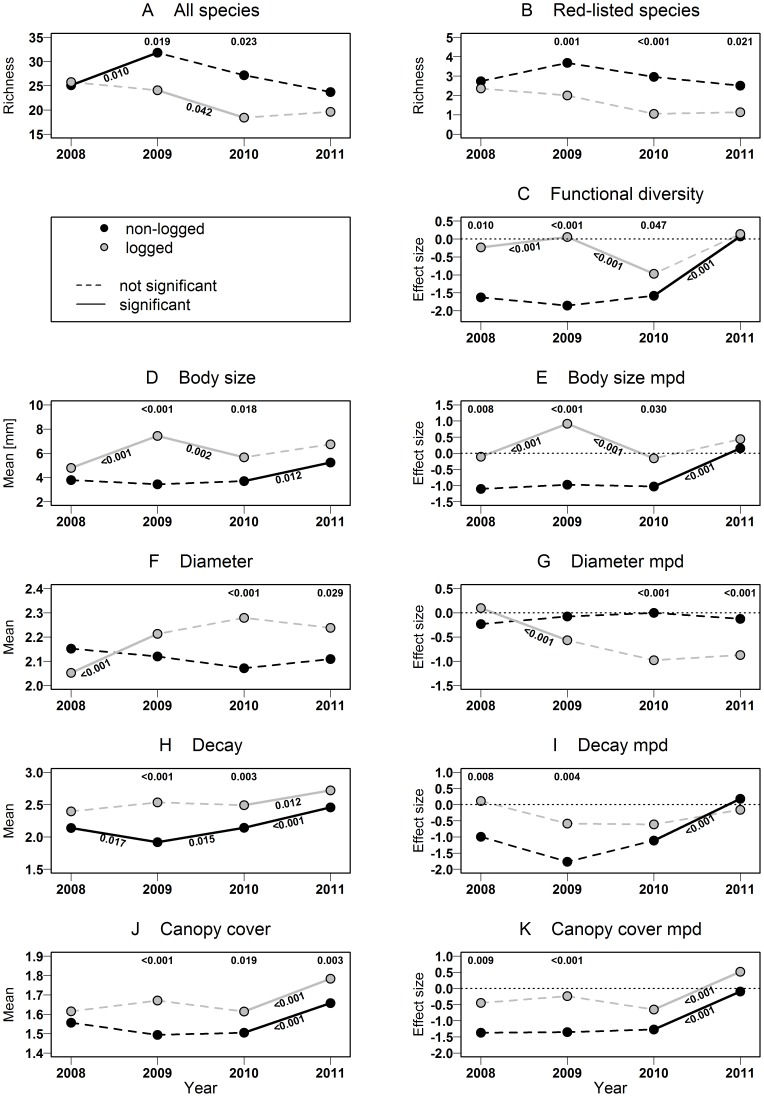
Effects of salvage logging on saproxylic beetles. Mean species richness of saproxylic beetles (A) and red-listed saproxylic beetles (B), abundance-weighted mean niche positions and standardized effect size (based on mean pairwise distance) of functional diversity (C) and body size (D, E), niche diameter (F, G), niche decay (H, I) and niche canopy cover (J, K) in logged and non-logged windthrow areas in a spruce mountain forest based on a GLMM with treatment and year as fixed factors, and space and plot as random factors. For multiple comparisons between treatments, the adjusted p-values are drawn above the respective points; for comparisons between different years within the same treatment, p-values are drawn below respective lines. Full details on p-values and model estimators can be found in Tables S2 and S3.

Salvage logging significantly decreased the mean number of the primary target species *I. typographus* per trap in 2008, 2009 and 2010. The mean number of *I*. *typographus* individuals significantly increased from 2008 to 2009 and significantly decreased from 2009 to 2010, reflecting the colonization of the windthrow by this pest species. The species richness of saproxylic beetles was significantly lower on salvage-logged plots than on non-logged plots in 2009 and 2010 (Fig. 2A). On non-logged plots, species richness significantly increased from 2008 to 2009, whereas on logged plots, richness significantly decreased from 2009 to 2010 (Fig. 2A). A similar trend was found only for red-listed species, which displayed significantly lower species richness on salvage-logged plots in 2009, 2010 and 2011 (Fig. 2B; for complete species list, see Table S1).

### Assemblage functional response

Beetle assemblages of logged and non-logged windthrows differed in their functional trait dispersion. The total functional diversity was lower on non-logged plots and revealed a clustered pattern, but was random on logged plots (Fig. 2C). From 2010 to 2011, the functional assemblage composition of non-logged plots developed significantly toward more random assemblages. The mean body sizes and the corresponding niche diversity were consistently and significantly higher on logged plots than on non-logged plots in 2009 and 2010 (Fig.2 D, E). We also found significantly higher mean diameter niche values on logged plots in 2010 and 2011, but not in 2008 and 2009 (Fig. 2F). The corresponding niche diversity of assemblages was significantly more randomly distributed on non-logged plots in 2010 and 2011 (Fig. 2G). In 2009 and 2010, the mean decay niche, which reflects the process of decomposition, was significantly higher on logged plots (Fig. 2H). The corresponding decay niche diversity (Fig. 2I) showed a significantly more clustered pattern on non-logged plots than on logged plots in 2008 and 2009. The mean canopy niche value of the local assemblages of logged and non-logged plots differed in 2009–2011. Significantly more species preferring open-canopy conditions (sunny habitats) were found on non-logged plots, as indicated by a significantly higher mean niche value of the canopy niche in 2009, 2010 and 2011 (Fig. 2J). A similar trend was found in the corresponding niche diversity, which displayed a significantly clustered pattern in the canopy cover niche in 2008 and 2009 (Fig. 2K). Full details on p-values and model estimators can be found in Tables S2 and S3.

## Discussion

Our study confirmed long-held empirical and scientific findings that salvage logging after windthrows reduces populations of pest bark beetles [7], [12] and has accompanying negative effects on the species richness of saproxylic beetles [51]. Surprisingly, our examination of the functional traits did not support most of our predictions: single-trait analyses revealed species on logged plots that were on average larger, preferred dead wood of larger diameter, and were adapted to shady habitats, compared to species on non-logged plots. These unexpected results underline the high sensitivity of an analysis based on species functional traits to detect complex and subtle changes in species assemblages caused by anthropogenic impact [26], [52]–[54].

Salvage logging aims at controlling populations of one or a few pest species but reduces biodiversity per se as collateral damage [6], [55], [56]. Accordingly, our results demonstrated that salvage logging dramatically decreases species richness of saproxylic beetles, including red-listed species and not only the target species *I. typographus* (Fig. 2A, B). These reductions seem to be mainly caused by the loss of species directly associated with *I. typographus* (compare [57]), e.g. by the decrease in predators of *I. typographus*, such as *Thanasimus* sp.; by the decrease in species that exploit bark beetle galleries, such as *Crypturgus cinereus*; and by the loss of species associated with a similar early decay stage of wood. Saint-Germain *et al.* [58] demonstrated that the majority of saproxylic beetle species on the conifer black spruce (*Picea mariana*) colonize the early decay stages, while saproxylic beetle species on the broadleaf tree aspen (*Populus tremula*) occur mostly on wood of later decay stages — a commonly observed pattern [41], [59], [60]. Our study, which focuses on the first four years of the decay process, therefore reflects the most important stage of succession within windthrown stands of coniferous trees [61]. In particular, because colonization patterns of saproxylic organisms this stage determines subsequent saproxylic communities, like.g. the early-arriving bark beetle *Hylurgops palliates* and the wood-decaying fungus *Fomitopsis pinicola*, enabled a higher colonization success of the endangered beetle *Peltis grossa* after 10 years [62].

### Larger beetles on logged plots

In contrast to our prediction, our results showed a consistent separation of beetle assemblages, from on average large species on logged plots toward small species on non-logged plots. One explanation for this pattern may be found behind the functional structure of the assemblages, i.e. in the habitat specificity of the species in our study: the majority of the larger species identified are widely distributed habitat generalists (except *Ampedus auripes*; [63]). One of the largest species, *Hylobius abietis*, which was more frequent in logged stands (see Table S1), is a known pest species attracted by the odour of resin in tree stumps [64]. Such species breed well in stumps and in logging residuals. Hence, an increase in the harvesting of stumps for bioenergy might expand the negative impacts of salvage logging to include currently less-affected species [65]–[67].

### Species preferring wood of large diameters on logged plots

After regular clear-cutting, the remaining dead wood amounts to approximately 10 m^3^ ha^−1^ [68]. Post-windthrow salvage-logged sites offer much more dead wood, e.g. from 45 to >70 m^3^ ha^−1^ on 90 sites in Switzerland [37]. Such high amounts of remaining dead wood on salvage-logged areas regularly surpass the critical thresholds of dead-wood amount for diversity in boreal forests [32]. Accordingly, the mean diameter niche position of beetles in our study was significantly higher in 2010 and 2011. Thereby, simply the amount of dead wood does not seem to be the limiting factor for saproxylic beetles species richness in salvage-logged plots. The limiting factor is rather the loss of small branches, as indicated by the loss of species preferring wood of small diameter (Fig. 2J). Accordingly, an alteration of the remaining dead-wood resources appears to be more crucial than the simple removal of the main wood volume by salvage logging.

This assumption is strongly supported by our findings on functional trait diversity (Fig. 2C): we found a clustering of functional assemblage structure on non-logged plots, which indicated a strong habitat-filtering (dead-wood resources) effect on dead-wood communities. Using co-occurrence null-model approaches, Azeria *et al*. [69] also found a strong habitat-filtering effect on saproxylic assemblages on burned trees. In accordance, Ding *et al.* [70] proposed that disturbance in forest ecosystems generates communities by abiotic filtering. Hence, our data suggested that anthropogenic intervention, i.e. salvage logging, of natural disturbances can disrupt the natural habitat filtering in the assembly process.

### Accelerated decomposition on salvage-logged plots

The amount and diversity of dead-wood resources of salvage-logged areas does not significantly differ between salvage-logged and non-salvage-logged windthrows [37]. But, in contrast to natural windthrows, the remaining dead wood on salvage-logged windthrows in our study area is mostly scattered on the ground surface (e.g. Fig. 1). Owing to the stronger attraction of wood-inhabiting fungi, salvage-logged sites tend to harbour more advanced decomposition stages than non-salvage-logged sites [37], [71], [72]. This shift within decay stages of available dead-wood resources was well reflected by our finding of a mean decay niche with significantly higher mean decay niche values on salvage-logged plots in 2009 and 2010. Furthermore, the corresponding niche diversity indicated a strong habitat filtering effect towards species of early decay stages on non-salvage-logged plots.

### Decrease of heliophilous species through salvage logging

Sun exposure increases the probability of the presence of red-listed species in aspen retention trees in clear-cuts in Norway [38]. Similarly, the endangered longhorn beetle *Rosalia alpina* prefers trees with a lower percentage of canopy closure and higher sun exposure than the average tree [73]. Hence, sun exposure is a major predictor determining saproxylic beetle communities in dead-wood resources [74], [75]. In our study, we demonstrated a shift of assemblages comprising heliophilous species on non-logged plots toward species preferring shady habitats on logged plots. This result is contrasts our prediction that the removal of dead wood might decrease the shady conditions provided by the cross-laminated arrangement of trees after windthrow. However, the majority of logging residuals on logged plots lie on the ground surface and are covered by an extensive grass layer, both of which create a moist microclimate, which decreases the availability of sun-exposed dead wood. An accelerated growth of natural regeneration on salvage-logged plots [76] does not appear to be of great importance, since natural regeneration in our study area is still poor and not able to shade the complete surroundings of a flight-interception trap (see Fig. 1 insets). Furthermore, Priewasser [77] demonstrated for a comparable study area that local factor, such as soil pH, are the main predictors for the growth of natural regeneration and not salvage logging per se. Based on our findings, it seems necessary to experimentally test our assumptions of the importance of microclimate and to estimate the amount of retention trees in windthrows sufficient for conserving saproxylic biodiversity.

## Conclusion

Spruce forests in Europe will be affected heavily by increasing storm damages in the future, which will lead to increasingly heated debates between economists and ecologists on the appropriate means to limit the negative effects of salvage logging on biodiversity [3], [4]. Our analysis based on functional traits revealed an unexpected response of saproxylic beetles to salvage logging and suggested that microclimate conditions are more crucial for the use of dead-wood resources by saproxylic beetles than dead-wood diameter or diversity [32], [37]. The direct relationship between species traits and logging-affected structures enables us to derive new guidelines for conservationists and managers to optimize salvage logging with a consideration of biodiversity conservation: downed tree tops unaffected by salvage logging operations and complete single windthrown trees should be preserved and allowed to naturally decay on salvage-logged areas to help sustain heliophilous species, colonizers of early decay stages and species that prefer wood of small diameter. Such a preservation of some windthrown trees in salvage-logged areas is an extension of the “green-tree retention approach” to downed trees in forests worldwide.

## Supporting Information

Table S1 **Total number of saproxylic beetles in logged and non-logged windthrown areas.**
(DOCX)

Table S2 **p-values based on a linear mixed effect model.**
(DOCX)

Table S3 **Model estimates and standard deviation.**
(DOCX)

Text S1 **R code used for general linear mixed model.**
(R) Click here for additional data file.
