# Supplementary material for: New Insights into the Consequences of Post-Windthrow Salvage Logging Revealed by Functional Structure of Saproxylic Beetles Assemblages
Source: PLoS One. 2014 Jul 22;9(7):e101757. doi: 10.1371/journal.pone.0101757 (PMC4106782; doi:10.1371/journal.pone.0101757)
Supplement: Table S1 — Total number of saproxylic beetles in logged and non-logged windthrown areas. (DOCX) [file pone.0101757.s001.docx]

**Table S1.** Red-list status, total number and niche positions of saproxylic beetles on logged and non-logged windthrow areas in the Bavarian Forest National Park.

| Family* | Species | Red list status** | Individuals | |  | Niches | | | |
| --- | --- | --- | --- | --- | --- | --- | --- | --- | --- |
|  |  |  | Logged | Non-logged |  | Canopy cover | Decay stage | Diameter | Body size [mm] |
| Histeridae | *Plegaderus vulneratus* (Panz., 1797) |  | 0 | 1 |  | 1.5 | 2 | 2.29 | 1 |
| Sphaeritidae | *Sphaerites glabratus* (F., 1792) |  | 0 | 1 |  | 2 | 3.2 | 2.5 | 6 |
| Leiodidae | *Anisotoma humeralis* (F., 1792) |  | 2 | 1 |  | 2.4 | 4 | 2.5 | 3 |
|  | *Anisotoma castanea* (Hbst., 1792) |  | 2 | 0 |  | 2.4 | 4 | 2.5 | 3 |
|  | *Agathidium nigripenne* (F., 1792) |  | 0 | 7 |  | 2 | 3 | 2 | 3 |
| Staphylinidae | *Phyllodrepa ioptera* (Steph., 1834) |  | 1 | 0 |  | 2.4 | 3.71 | 3 | 2 |
|  | *Phloeonomus pusillus* (Grav., 1806) |  | 0 | 1 |  | 1.5 | 2.5 | 2.33 | 1 |
|  | *Nudobius lentus* (Grav., 1806) |  | 17 | 43 |  | 1.6 | 2.25 | 2.6 | 7 |
|  | *Atrecus affinis* (Payk., 1789) |  | 4 | 1 |  | 2.6 | 4 | 2.6 | 6 |
|  | *Quedius maurus* (Sahlb., 1830) |  | 0 | 1 |  | 2.17 | 4.6 | 2.33 | 7 |
|  | *Quedius plagiatus* (Mannh., 1843) | 3 | 0 | 25 |  | 2.4 | 3.67 | 2.33 | 7 |
|  | *Placusa depressa* (Maekl., 1845) |  | 7 | 10 |  | 1.5 | 2.25 | 2.6 | 2 |
|  | *Placusa tachyporoides* (Waltl, 1838) |  | 0 | 1 |  | 1.6 | 1.6 | 2.6 | 2 |
|  | *Homalota plana* (Gyll., 1810) |  | 0 | 1 |  | 1.83 | 1.75 | 2.6 | 2 |
|  | *Leptusa pulchella* (Mannh., 1830) |  | 2 | 8 |  | 1.83 | 3.8 | 2.6 | 2 |
|  | *Leptusa fumida* (Er., 1839) |  | 5 | 5 |  | 2.5 | 3.8 | 2.6 | 2 |
|  | *Phloeopora corticalis* (Grav., 1802) |  | 1 | 0 |  | 1.5 | 2.25 | 2 | 2 |
| Pselaphidae | *Bibloporus bicolor* (Denny, 1825) |  | 2 | 1 |  | 2.4 | 3.4 | 3 | 1 |
|  | *Plectophloeus fischeri* (Aube, 1833) |  | 4 | 0 |  | 2.5 | 4.17 | 3 | 1 |
| Lycidae | *Dictyopterus aurora* (Hbst., 1784) |  | 3 | 3 |  | 2.4 | 4 | 2.5 | 10 |
|  | *Pyropterus nigroruber* (DeGeer, 1774) |  | 1 | 1 |  | 1.5 | 4 | 2.5 | 8 |
|  | *Platycis minutus* (F., 1787) |  | 0 | 1 |  | 2.4 | 4 | 2.5 | 7 |
| Cantharidae | *Malthodes fuscus* (Waltl, 1838) |  | 9 | 7 |  | 1.67 | 3.2 | 2.29 | 4 |
|  | *Malthodes alpicola* (Kiesw., 1852) | R | 21 | 27 |  | 1.67 | 3.2 | 2.29 | 4 |
|  | *Malthodes guttifer* (Kiesw., 1852) |  | 1 | 0 |  | 1.67 | 3.2 | 2.29 | 4 |
|  | *Malthodes hexacanthus* (Kiesw., 1852) |  | 2 | 2 |  | 1.67 | 3.2 | 2.29 | 2 |
| Melyridae | *Aplocnemus nigricornis* (F., 1792) |  | 2 | 1 |  | 1.5 | 2.5 | 2.29 | 4 |
|  | *Aplocnemus tarsalis* (Sahlb., 1822) | 3 | 15 | 35 |  | 1.5 | 2.5 | 2.29 | 5 |
|  | *Dasytes niger* (L., 1761) |  | 148 | 62 |  | 1 | 3.2 | 2.29 | 4 |
|  | *Dasytes obscurus* (Gyll., 1813) |  | 172 | 90 |  | 1.5 | 3.2 | 1.86 | 5 |
|  | *Dasytes plumbeus* (Müll., 1776) |  | 8 | 3 |  | 1.5 | 3.2 | 1.86 | 4 |
| Cleridae | *Thanasimus formicarius* (L., 1758) |  | 23 | 36 |  | 1.5 | 2.25 | 2.29 | 8 |
|  | *Thanasimus pectoralis* (Fuss, 1863) | D | 19 | 38 |  | 1.4 | 2.25 | 2.29 | 6 |
| Trogossitidae | *Nemosoma elongatum* (L., 1761) |  | 10 | 48 |  | 1.4 | 2 | 2 | 5 |
| Peltidae | *Ostoma ferruginea* (L., 1758) | 3 | 4 | 0 |  | 1.4 | 3.4 | 3 | 8 |
|  | *Thymalus limbatus* (F., 1787) | 3 | 0 | 1 |  | 2 | 3.25 | 2.5 | 6 |
| Lymexylonidae | *Hylecoetus dermestoides* (L., 1761) |  | 67 | 110 |  | 1.5 | 2 | 3 | 12 |
| Elateridae | *Ampedus erythrogonus* (Müll., 1821) | 3 | 5 | 5 |  | 2.5 | 3.86 | 3.5 | 6 |
|  | *Ampedus balteatus* (L., 1758) |  | 52 | 15 |  | 1.5 | 3.4 | 2.5 | 8 |
|  | *Ampedus aethiops* (Lacord., 1835) |  | 94 | 88 |  | 1.5 | 3.4 | 2.5 | 10 |
|  | *Ampedus sanguineus* (L., 1758) |  | 1 | 0 |  | 1.5 | 3.4 | 3 | 14 |
|  | *Ampedus melanurus* (Muls.Guillb., 1855) | 1 | 3 | 2 |  | 1.5 | 3.4 | 3 | 8 |
|  | *Ampedus nigrinus* (Hbst., 1784) |  | 480 | 189 |  | 2.5 | 3.67 | 2.5 | 8 |
|  | *Ampedus auripes* (Reitter, 1895) | G | 155 | 252 |  | 1.5 | 3.4 | 3 | 9 |
|  | *Melanotus castanipes* (Payk., 1800) |  | 100 | 29 |  | 2.4 | 3.67 | 3 | 17 |
|  | *Anostirus purpureus* (Poda, 1761) |  | 0 | 1 |  | 1.4 | 3.4 | 2.5 | 11 |
|  | *Anostirus castaneus* (L., 1758) |  | 4 | 5 |  | 1.4 | 3.4 | 2.5 | 9 |
|  | *Anostirus sulphuripennis* (Germ., 1843) | 3 | 2 | 0 |  | 1.4 | 3.4 | 2.5 | 11 |
|  | *Denticollis rubens* (Pill.Mitt., 1783) | 2 | 7 | 10 |  | 2.6 | 3.4 | 2.29 | 13 |
|  | *Diacanthous undulatus* (DeGeer, 1774) | R | 7 | 24 |  | 2.5 | 3.4 | 3 | 15 |
| Buprestidae | *Anthaxia helvetica* (Stierl., 1868) |  | 28 | 29 |  | 1 | 2 | 1.25 | 6 |
|  | *Anthaxia quadripunctata* (L., 1758) |  | 5 | 8 |  | 1 | 1.75 | 1.2 | 6 |
| Dermestidae | *Megatoma undata* (L., 1758) | 3 | 2 | 0 |  | 1.5 | 3.5 | 3 | 5 |
| Cerylonidae | *Cerylon ferrugineum* (Steph., 1830) |  | 1 | 1 |  | 1.5 | 2.4 | 2.5 | 1 |
| Nitidulidae | *Epuraea deubeli* (Rtt., 1898) |  | 3 | 5 |  | 1.5 | 2 | 3 | 2 |
|  | *Epuraea thoracica* (Tourn., 1872) | 3 | 19 | 96 |  | 1.5 | 2 | 1.8 | 3 |
|  | *Epuraea angustula* (Sturm, 1844) | 3 | 2 | 5 |  | 1.4 | 2 | 2.5 | 2 |
|  | *Epuraea boreella* (Zett., 1828) | 3 | 9 | 35 |  | 1.5 | 2 | 2 | 2 |
|  | *Epuraea marseuli* (Rtt., 1872) |  | 20 | 64 |  | 1.5 | 2.25 | 2.2 | 3 |
|  | *Epuraea pygmaea* (Gyll., 1808) |  | 31 | 50 |  | 1.5 | 2 | 2 | 2 |
|  | *Epuraea binotata* (Rtt., 1872) |  | 13 | 46 |  | 1.5 | 2 | 2.2 | 2 |
|  | *Epuraea terminalis* (Mannh., 1843) |  | 0 | 1 |  | 1.6 | 1.75 | 2 | 3 |
|  | *Epuraea variegata* (Hbst., 1793) |  | 4 | 12 |  | 2.5 | 3.4 | 2.5 | 2 |
|  | *Epuraea rufomarginata* (Steph., 1830) |  | 4 | 15 |  | 1.6 | 2 | 1.2 | 3 |
|  | *Ipidia binotata* (Rtt., 1875) | 1 | 8 | 0 |  | 1.5 | 2.5 | 3 | 4 |
|  | *Cychramus variegatus* (Hbst., 1792) |  | 1 | 0 |  | 2 | 3.4 | 3 | 6 |
|  | *Cychramus luteus* (F., 1787) |  | 0 | 1 |  | 2 | 3.4 | 2.5 | 4 |
|  | *Glischrochilus quadripunctatus* (L., 1758) |  | 11 | 13 |  | 1.5 | 2 | 2.29 | 4 |
|  | *Pityophagus ferrugineus* (L., 1761) |  | 18 | 19 |  | 1.5 | 2 | 2.5 | 5 |
| Monotomidae | *Rhizophagus depressus* (F., 1792) |  | 0 | 14 |  | 1.4 | 2 | 2.5 | 3 |
|  | *Rhizophagus ferrugineus* (Payk., 1800) |  | 109 | 162 |  | 2 | 2 | 2.5 | 3 |
|  | *Rhizophagus dispar* (Payk., 1800) |  | 8 | 18 |  | 1.6 | 2.5 | 2.5 | 3 |
|  | *Rhizophagus bipustulatus* (F., 1792) |  | 12 | 41 |  | 1.6 | 2.5 | 2.5 | 2 |
|  | *Rhizophagus nitidulus* (F., 1798) | 3 | 4 | 1 |  | 2 | 2.8 | 2.29 | 3 |
| Erotylidae | *Triplax aenea* (Schall., 1783) | 3 | 0 | 1 |  | 1.5 | 3.4 | 3 | 3 |
|  | *Triplax russica* (L., 1758) | 3 | 2 | 0 |  | 2.17 | 3.4 | 3 | 5 |
| Cryptophagidae | *Pteryngium crenatum* (F., 1798) | 3 | 5 | 6 |  | 2 | 3 | 2.5 | 1 |
|  | *Cryptophagus cylindrus* (Kiesw., 1858) |  | 0 | 1 |  | 1 | 2 | 1 | 1 |
|  | *Micrambe abietis* (Payk., 1798) |  | 2 | 1 |  | 1.5 | 2 | 1 | 2 |
|  | *Atomaria ornata* (Heer, 1841) |  | 3 | 3 |  | 2.25 | 2 | 1 | 1 |
|  | *Atomaria turgida* (Er., 1846) |  | 7 | 6 |  | 2 | 2 | 1 | 1 |
|  | *Atomaria diluta* (Er., 1846) | 3 | 1 | 1 |  | 2.5 | 3.29 | 2 | 1 |
| Latridiidae | *Latridius hirtus* (Gyll., 1827) | 3 | 3 | 0 |  | 2.17 | 4 | 2.2 | 1 |
|  | *Stephostethus alternans* (Mannh., 1844) |  | 57 | 96 |  | 2.4 | 3 | 1.5 | 2 |
|  | *Corticaria interstitialis* (Mannh., 1844) |  | 0 | 1 |  | 2.5 | 3 | 2.2 | 1 |
|  | *Corticaria abietorum* (Motsch., 1867) | 3 | 46 | 74 |  | 2 | 2.25 | 1 | 1 |
| Mycetophagidae | *Litargus connexus* (Fourcr., 1785) |  | 1 | 0 |  | 1.5 | 2.75 | 2.29 | 2 |
| Endomychidae | *Mycetina cruciata* (Schall., 1783) | 2 | 4 | 0 |  | 2.4 | 3.5 | 3 | 4 |
|  | *Endomychus coccineus* (L., 1758) |  | 1 | 0 |  | 2.17 | 3.4 | 2.29 | 5 |
| Cisidae | *Octotemnus glabriculus* (Gyll., 1827) |  | 0 | 1 |  | 2.5 | 3.4 | 2.5 | 1 |
|  | *Cis hispidus* (Payk., 1798) |  | 0 | 1 |  | 1.67 | 3.25 | 2.5 | 2 |
|  | *Cis boleti* (Scop., 1763) |  | 2 | 0 |  | 1.75 | 3.4 | 2.5 | 3 |
|  | *Cis punctulatus* (Gyll., 1827) |  | 6 | 6 |  | 1.5 | 3.25 | 2.2 | 2 |
|  | *Cis dentatus* (Mell., 1848) |  | 2 | 7 |  | 1.5 | 3.25 | 2.5 | 2 |
|  | *Orthocis festivus* (Panz., 1793) |  | 2 | 3 |  | 1.75 | 3 | 1.86 | 2 |
|  | *Hadreule elongatulum* (Gyll., 1827) | G | 2 | 0 |  | 1 | 3.43 | 3 | 1 |
| Anobiidae | *Hedobia imperialis* (L., 1767) |  | 0 | 1 |  | 1.5 | 3 | 1.86 | 4 |
|  | *Xestobium austriacum* (Rtt., 1890) | 2 | 1 | 2 |  | 1.8 | 3 | 2.29 | 7 |
|  | *Ernobius abietis* (F., 1792) |  | 28 | 47 |  | 1.5 | 1.86 | 1 | 3 |
|  | *Anobium emarginatum* (Duft., 1825) | 3 | 2 | 4 |  | 1.5 | 1.8 | 2.2 | 4 |
|  | *Anobium pertinax* (L., 1758) |  | 1 | 0 |  | 1.5 | 3 | 3 | 5 |
|  | *Ptilinus pectinicornis* (L., 1758) |  | 1 | 2 |  | 1.5 | 3 | 2.5 | 4 |
| Oedemeridae | *Calopus serraticornis* (L., 1758) |  | 3 | 1 |  | 2.5 | 3.4 | 2.29 | 19 |
|  | *Chrysanthia viridissima* (L., 1758) |  | 2 | 0 |  | 1.5 | 3.4 | 1.2 | 8 |
|  | *Chrysanthia nigricornis* (Westh., 1882) |  | 1 | 0 |  | 1.5 | 3.4 | 1.2 | 6 |
| Pythidae | *Pytho depressus* (L., 1767) |  | 0 | 1 |  | 1.5 | 3 | 2.33 | 11 |
| Salpingidae | *Salpingus ruficollis* (L., 1761) |  | 6 | 6 |  | 2 | 2.25 | 2 | 3 |
| Scraptiidae | *Anaspis ruficollis* (F., 1792) | 2 | 4 | 6 |  | 1.5 | 4 | 2.29 | 2 |
|  | *Anaspis rufilabris* (Gyll., 1827) |  | 175 | 550 |  | 1.5 | 4 | 2.29 | 2 |
| Mordellidae | *Curtimorda maculosa* (Naez., 1794) | 3 | 21 | 33 |  | 1 | 3.5 | 3 | 4 |
| Melandryidae | *Hallomenus binotatus* (Quensel, 1790) |  | 4 | 0 |  | 2 | 3.4 | 2.5 | 4 |
|  | *Orchesia micans* (Panz., 1794) |  | 3 | 0 |  | 2.17 | 3.25 | 2.5 | 4 |
|  | *Orchesia undulata* (Kr., 1853) |  | 0 | 2 |  | 2.5 | 3.5 | 2.5 | 4 |
|  | *Abdera flexuosa* (Payk., 1799) | 3 | 0 | 5 |  | 2.4 | 3.25 | 2.29 | 3 |
|  | *Xylita laevigata* (Hell., 1786) | 2 | 4 | 0 |  | 2 | 3.5 | 2.5 | 8 |
|  | *Xylita livida* (Sahlb., 1834) | 2 | 19 | 20 |  | 1.5 | 3.5 | 2.5 | 5 |
|  | *Serropalpus barbatus* (Schall., 1783) |  | 3 | 2 |  | 2 | 2.25 | 2.2 | 13 |
| Tenebrionidae | *Corticeus linearis* (F., 1790) |  | 12 | 22 |  | 1.4 | 2 | 1 | 2 |
| Scarabaeidae | *Trichius fasciatus* (L., 1758) |  | 3 | 1 |  | 1.5 | 3.5 | 2.5 | 10 |
| Lucanidae | *Platycerus caprea* (DeGeer, 1774) |  | 36 | 9 |  | 2 | 3.4 | 2 | 14 |
| Cerambycidae | *Tetropium castaneum* (L., 1758) |  | 121 | 92 |  | 1.5 | 2 | 2.2 | 13 |
|  | *Tetropium fuscum* (F., 1758) |  | 4 | 9 |  | 1.5 | 2 | 2.2 | 12 |
|  | *Rhagium bifasciatum* (F., 1775) |  | 279 | 345 |  | 2.5 | 3.4 | 2.5 | 17 |
|  | *Rhagium mordax* (DeGeer, 1775) |  | 7 | 12 |  | 1.5 | 2.25 | 2.5 | 17 |
|  | *Rhagium inquisitor* (L., 1758) |  | 36 | 16 |  | 1.8 | 2.4 | 2.5 | 15 |
|  | *Oxymirus cursor* (L., 1758) |  | 129 | 47 |  | 1.8 | 3.6 | 2.5 | 23 |
|  | *Evodinus clathratus* (F., 1792) |  | 19 | 35 |  | 1.5 | 3 | 1.8 | 11 |
|  | *Gaurotes virginea* (L., 1758) |  | 23 | 8 |  | 1.5 | 3.4 | 2.29 | 10 |
|  | *Corymbia maculicornis* (DeGeer, 1775) |  | 4 | 0 |  | 1.8 | 3.5 | 2.5 | 9 |
|  | *Corymbia rubra* (L., 1758) |  | 29 | 7 |  | 1.5 | 3.5 | 2.5 | 14 |
|  | *Anastrangalia sanguinolenta* (L., 1761) |  | 15 | 5 |  | 1.5 | 3.5 | 2.29 | 10 |
|  | *Anastrangalia dubia* (Scop., 1763) |  | 2 | 1 |  | 1.5 | 3 | 2.5 | 12 |
|  | *Lepturobosca virens* (L., 1758) | 3 | 1 | 0 |  | 1.5 | 3.5 | 2.5 | 18 |
|  | *Judolia sexmaculata* (L., 1758) | 2 | 63 | 39 |  | 2.5 | 3.4 | 2.29 | 11 |
|  | *Pachytodes cerambyciformis* (Schrk., 1781) |  | 1 | 1 |  | 1.8 | 3.17 | 2.29 | 9 |
|  | *Stenurella melanura* (L., 1758) |  | 78 | 36 |  | 1.5 | 3.4 | 1 | 7 |
|  | *Molorchus minor* (L., 1758) |  | 42 | 56 |  | 1.5 | 2 | 1.2 | 11 |
|  | *Callidium coriaceum* (Payk., 1800) | 3 | 1 | 0 |  | 1.4 | 2 | 2 | 11 |
|  | *Callidium aeneum* (DeGeer, 1775) |  | 0 | 2 |  | 1.4 | 2 | 1.25 | 12 |
|  | *Clytus lama* (Muls., 1847) |  | 15 | 0 |  | 1.4 | 2 | 1.5 | 11 |
|  | *Monochamus sutor* (L., 1758) |  | 2 | 5 |  | 1 | 2 | 2 | 19 |
|  | *Pogonocherus fasciculatus* (DeGeer, 1775) |  | 17 | 15 |  | 1.5 | 2 | 1 | 6 |
|  | *Pogonocherus ovatus* (Goeze, 1777) | 1 | 1 | 0 |  | 2 | 2.25 | 1 | 5 |
|  | *Acanthocinus griseus* (F., 1792) | 3 | 0 | 2 |  | 1.5 | 2 | 1.25 | 10 |
| Scolytidae | *Phthorophloeus spinulosus* (Rey, 1883) |  | 13 | 59 |  | 1.4 | 2 | 1 | 2 |
|  | *Hylastes cunicularius* (Er., 1836) |  | 1101 | 1251 |  | 1.5 | 2 | 2.5 | 3 |
|  | *Hylastes attenuatus* (Er., 1836) |  | 0 | 3 |  | 1.5 | 2 | 1.8 | 2 |
|  | *Hylurgops glabratus* (Zett., 1828) |  | 14 | 24 |  | 2.2 | 2 | 2.5 | 4 |
|  | *Hylurgops palliatus* (Gyll., 1813) |  | 37 | 136 |  | 2.2 | 2 | 2.5 | 2 |
|  | *Dendroctonus micans* (Kug., 1794) |  | 1 | 0 |  | 1 | 2 | 2.6 | 8 |
|  | *Polygraphus grandiclava* (Thoms., 1886) |  | 0 | 7 |  | 1.5 | 2 | 1.2 | 2 |
|  | *Polygraphus poligraphus* (L., 1758) |  | 39 | 220 |  | 1.5 | 2 | 1.8 | 2 |
|  | *Leperisinus fraxini* (Panz., 1799) |  | 1 | 0 |  | 1.5 | 2 | 2.71 | 3 |
|  | *Xylechinus pilosus* (Ratz., 1837) |  | 5 | 106 |  | 1.4 | 2 | 2.17 | 2 |
|  | *Crypturgus cinereus* (Aubé, 1862) |  | 18 | 59 |  | 2 | 2 | 2.2 | 1 |
|  | *Crypturgus hispidulus* (Thoms., 1870) |  | 15 | 10 |  | 2 | 2 | 2.2 | 1 |
|  | *Dryocoetes autographus* (Ratz., 1837) |  | 438 | 931 |  | 1.6 | 2 | 2.4 | 3 |
|  | *Dryocoetes hectographus* (Rtt., 1913) |  | 133 | 290 |  | 1.6 | 2 | 2 | 3 |
|  | *Cryphalus abietis* (Ratz., 1837) |  | 25 | 109 |  | 1 | 2 | 1 | 1 |
|  | *Ernoporicus fagi* (F., 1778) |  | 1 | 0 |  | 1.5 | 2 | 1 | 1 |
|  | *Pityophthorus pityographus* (Ratz., 1837) |  | 62 | 645 |  | 1.5 | 1.75 | 1 | 1 |
|  | *Taphrorychus bicolor* (Hbst., 1793) |  | 2 | 0 |  | 1.5 | 2 | 1.8 | 2 |
|  | *Pityogenes chalcographus* (L., 1761) |  | 3147 | 6697 |  | 1.4 | 1.75 | 1.67 | 2 |
|  | *Pityogenes conjunctus* (Rtt., 1887) |  | 112 | 289 |  | 1 | 2 | 1 | 2 |
|  | *Pityogenes bidentatus* (Hbst., 1783) |  | 11 | 9 |  | 1.4 | 2 | 1 | 2 |
|  | *Orthotomicus laricis* (F., 1792) |  | 9 | 8 |  | 1.5 | 2 | 1.8 | 3 |
|  | *Ips typographus* (L., 1758) |  | 796 | 7785 |  | 1.5 | 1.75 | 2.5 | 4 |
|  | *Ips amitinus* (Eichh., 1871) |  | 74 | 428 |  | 1.4 | 2 | 1.75 | 4 |
|  | *Xyloterus domesticus* (L., 1758) |  | 4 | 13 |  | 1.6 | 2 | 2.5 | 3 |
|  | *Xyloterus lineatus* (Ol., 1795) |  | 208 | 1579 |  | 1.5 | 2 | 2.29 | 3 |
|  | *Xyloterus laevae* (Eggers, 1939) | 2 | 13 | 66 |  | 1.5 | 2 | 2.5 | 3 |
| Curculionidae | *Rhyncolus ater* (L., 1758) |  | 5 | 7 |  | 2.4 | 3 | 2.29 | 3 |
|  | *Pissodes validirostris* (Sahlb., 1834) | 2 | 0 | 1 |  | 1.4 | 1 | 1 | 5 |
|  | *Pissodes harcyniae* (Hbst., 1795) | 3 | 1 | 1 |  | 1.4 | 2 | 1.75 | 5 |
|  | *Magdalis nitida* (Gyll., 1827) |  | 16 | 19 |  | 1.4 | 2 | 1 | 4 |
|  | *Trachodes hispidus* (L., 1758) |  | 0 | 3 |  | 2.25 | 3.5 | 1 | 3 |
|  | *Hylobius piceus* (DeGeer, 1775) |  | 3 | 6 |  | 1.5 | 2 | 2.5 | 14 |
|  | *Hylobius abietis* (L., 1758) |  | 85 | 24 |  | 1.5 | 2 | 2.5 | 10 |
|  | *Acalles pyrenaeus* (Boh., 1844) |  | 1 | 0 |  | 2.5 | 3.5 | 1 | 3 |

* Family order refers to that in Schmidl and Bußler (2004)

** Red list status according to (Schmidl et al., 2003): 1, critically endangered; 2, endangered; 3, vulnerable; G, threat assumed; R, potentially threatened.

**References**

Schmidl, J. & Bußler, H. (2004) Ökologische Gilden xylobionter Käfer Deutschlands. *Naturschutz und Landschaftsplanung,* **36,** 202-218.

Schmidl, J., Bussler, H. & Lorenz, W. (2003) Die Rote Liste gefährdeter Käfer Bayerns im

Überblick. *Schriftenreihe Bayerisches Landesamt für Umweltschutz,* **166**.
