# Supplementary material for: New Insights into the Consequences of Post-Windthrow Salvage Logging Revealed by Functional Structure of Saproxylic Beetles Assemblages
Source: PLoS One. 2014 Jul 22;9(7):e101757. doi: 10.1371/journal.pone.0101757 (PMC4106782; doi:10.1371/journal.pone.0101757)
Supplement: Table S2 — p-values based on a linear mixed effect model. (DOCX) [file pone.0101757.s002.docx]

**Table S2** p-values based on a generalized linear mixed model for the number of species and red-listed species, functional diversity, mean niche positions (niche) and effect size of the mean pairwise distance of niche positions (e-size). Significant p-values are in boldface.

|  | Analysed years | Number of species | Red list species | Functional diversity | Niche diameter | | Niche decay | | Niche canopy cover | | Body size | |
| --- | --- | --- | --- | --- | --- | --- | --- | --- | --- | --- | --- | --- |
|  |  |  |  | e-size | Niche | e-size | Niche | e-size | Niche | e-size | Niche | e-size |
| Logged vs. non-logged | 2008 | 1.000 | 0.976 | **0.010** | 0.171 | 0.404 | 0.070 | **0.008** | 0.546 | **0.009** | 0.608 | **0.008** |
|  | 2009 | **0.019** | **0.001** | **<0.001** | 0.246 | 0.051 | **<0.001** | **0.004** | **<0.001** | **<0.001** | **<0.001** | **<0.001** |
|  | 2010 | **0.005** | **<0.001** | **0.047** | **<0.001** | **<0.001** | **0.003** | 0.655 | **0.019** | 0.192 | **0.018** | **0.030** |
|  | 2011 | 0.565 | **0.021** | **0.997** | **0.029** | **<0.001** | 0.064 | 0.930 | **0.003** | 0.195 | 0.142 | 0.946 |
| Logged | 2009 vs. 2008 | 0.974 | 0.919 | **<0.001** | **<0.001** | **<0.001** | 0.349 | 0.134 | 0.432 | 0.949 | **<0.001** | **<0.001** |
|  | 2010 vs. 2009 | **0.042** | 0.055 | **<0.001** | 0.537 | 0.117 | 0.996 | 1.000 | 0.424 | 0.402 | **0.002** | **<0.001** |
|  | 2011 vs. 2010 | 0.997 | 0.999 | 0.121 | 0.922 | 0.995 | **0.012** | 0.612 | **<0.001** | **<0.001** | 0.190 | 0.103 |
| Non-logged | 2009 vs. 2008 | **0.008** | 0.055 | 1.00 | 0.978 | 0.954 | **0.017** | 0.068 | 0.290 | 1.000 | 0.987 | 0.998 |
|  | 2010 vs. 2009 | 0.167 | 0.268 | 1.00 | 0.835 | 0.999 | **0.015** | 0.193 | 1.000 | 1.000 | 0.998 | 1.000 |
|  | 2011 vs. 2010 | 0.487 | 0.792 | **<0.001** | 0.948 | 0.987 | **<0.001** | **<0.001** | **<0.001** | **<0.001** | **0.012** | **<0.001** |
