# Supplementary material for: New Insights into the Consequences of Post-Windthrow Salvage Logging Revealed by Functional Structure of Saproxylic Beetles Assemblages
Source: PLoS One. 2014 Jul 22;9(7):e101757. doi: 10.1371/journal.pone.0101757 (PMC4106782; doi:10.1371/journal.pone.0101757)
Supplement: Table S3 — Model estimates and standard deviation. (DOCX) [file pone.0101757.s003.docx]

**Table S3** Estimates and standard deviation based on a generalized linear mixed model for the number of species and red-listed species, functional diversity, mean niche positions (niche) and effect size of the mean pairwise distance of niche positions (e-size).

|  | Analysed years | Number of species | Red list species | Functional diversity | Niche diameter | | | Niche decay | | Niche canopy cover | | | | Body size | | |
| --- | --- | --- | --- | --- | --- | --- | --- | --- | --- | --- | --- | --- | --- | --- | --- | --- |
|  |  |  |  | e-size | Niche | e-size | | Niche | e-size | Niche | e-size | | Niche | | e-size | |
| Logged vs. non-logged | 2008 | −5.50 ±4.26 | −0.36 ±0.45 | 1.01 ±0.31 | 0.26 ±0.10 | 0.33 ±0.18 | 0.26 ±0.10 | | 1.10 ±0.33 | 0.06 ±0.04 | | 0.92 ±0.28 | 1.00 ±0.64 | | | 0.99 ±0.30 |
|  | 2009 | −16.54 ±4.26 | −1.68 ±0.45 | 1.97 ±0.31 | 0.61 ±0.10 | −0.49 ±0.18 | 0.61 ±0.10 | | 1.18 ±0.33 | 0.18 ±0.04 | | 1.11 ±0.28 | 4.00 ±0.64 | | | 1.89 ±0.30 |
|  | 2010 | −12.95 ±4.26 | −1.91 ±0.45 | 0.87 ±0.31 | 0.35 ±0.10 | −0.98 ±0.18 | 0.35 ±0.10 | | 0.50 ±0.33 | 0.11 ±0.04 | | 0.62 ±0.28 | 1.97 ±0.64 | | | 0.87 ±0.30 |
|  | 2011 | −6.22 ±4.26 | −1.36 ±0.45 | 0.18 ±0.31 | 0.26 ±0.10 | −0.75 ±0.18 | 0.26 ±0.10 | | −0.34 ±0.33 | 0.13 ±0.04 | | 0.62 ±0.28 | 1.51 ±0.64 | | | 0.29 ±0.30 |
| Logged | 2009 vs. 2008 | 0.81 ±3.58 | −0.36 ±0.35 | 1.05 ±0.25 | 0.14 ±0.07 | −0.66 ±0.17 | 0.12 ±0.10 | | −0.70 ±0.29 | 0.06 ±0.03 | | 0.21 ±0.22 | 2.64 ±0.48 | | | 1.03 ±0.24 |
|  | 2010 vs. 2009 | −12.90 ±3.58 | −0.95 ±0.35 | −1.13 ±0.25 | −0.04 ±0.07 | −0.41 ±0.17 | −0.04 ±0.07 | | −0.03 ±0.29 | −0.06 ±0.03 | | −0.42 ±0.22 | −1.76 ±0.48 | | | −1.07 ±0.24 |
|  | 2011 vs. 2010 | 2.05 ±3.58 | 0.09 ±0.35 | 0.62 ±0.25 | 0.23 ±0.07 | 0.17 ±0.17 | 0.23 ±0.07 | | 0.46 ±0.29 | 0.17 ±0.03 | | 1.18 ±0.22 | 1.07 ±0.48 | | | 0.60 ±0.24 |
| Non-logged | 2009 vs. 2008 | 11.86 ±3.58 | 0.95 ±0.35 | 0.09 ±0.25 | −0.22 ±0.07 | 0.15 ±0.17 | −0.21 ±0.07 | | −0.77 ±0.29 | −0.06 ±0.03 | | 0.02 ±0.22 | −0.35 ±0.48 | | | 0.13 ±0.24 |
|  | 2010 vs. 2009 | −16.50 ±3.58 | −0.73 ±0.35 | −0.03 ±0.25 | 0.22 ±0.07 | 0.07 ±0.17 | 0.22 ±0.07 | | 0.65 ±0.29 | 0.01 ±0.03 | | 0.08 ±0.22 | 0.26 ±0.48 | | | −0.05 ±0.24 |
|  | 2011 vs. 2010 | −4.68 ±3.58 | −0.45 ±0.35 | 1.31 ±0.25 | 0.32 ±0.07 | −0.12 ±0.17 | 0.31 ±0.07 | | 1.30 ±0.30 | 0.15 ±0.03 | | 1.18 ±0.22 | 1.53 ±0.48 | | | 1.18 ±0.24 |
